# Towards eliminating hepatitis C as a public health threat: different speeds, different needs

**DOI:** 10.2807/1560-7917.ES.2024.29.30.2400462

**Published:** 2024-07-25

**Authors:** Mojca Matičič, Maria Buti

**Affiliations:** 1 Clinic for Infectious Diseases and Febrile Illnesses, University Medical Centre Ljubljana, Ljubljana, Slovenia; 2 Faculty of Medicine, University of Ljubljana, Ljubljana, Slovenia; 3 Liver Unit, Department of Medicine, Vall d’Hebron Hospital Universitari, Barcelona, Spain; 4 CIBERehd del Instituto de Salud Carlos III, Madrid, Spain

**Keywords:** hepatitis C, elimination, prevalence, people who inject drugs, linkage to care, SDG, sustainable development goals

A decade ago, the introduction of direct acting antivirals (DAAs) presented one of the most revolutionary scientific achievements in the control of hepatitis C. Today, DAAs can safely cure more than 97% of hepatitis C virus (HCV)-infected individuals. Already in 2016, the World Health Assembly (WHA) unanimously adopted the resolution that viral hepatitis should be eliminated by 2030. In addition, the World Health Organization (WHO) published the Global Health Sector Strategies on viral hepatitis raising the prospect of eliminating HCV infection as a public health threat by aligning with the Sustainable Development Goals (SDG). The WHO called for integrated global efforts and established a framework to guide implementation of the key interventions at the national level in order to decrease chronic HCV infection incidence by 90% and mortality due to HCV infection by 65%, thus controlling viral hepatitis by the year 2030 [1,2]. The World Hepatitis Day 2024 on 28 July is a welcome occasion to reflect on this.

The global burden of hepatitis C is significant. Each year, there are hundreds and thousands of deaths due to end-stage liver disease, and half a million deaths due to hepatocellular carcinoma (HCC) [3]. Hepatocellular carcinoma is most commonly caused by the carcinogenic effect of chronic HCV infection. If DAA treatment is started late, i.e. when advanced liver fibrosis has developed, we have lost momentum, since in that case the risk for developing HCC after curing HCV infection persists, and therefore a lifelong HCC screening is warranted [4]. Despite HCC being the only form of cancer with an increased incidence over the last 10 years, a recommendation for the early detection of HCV infection and its immediate cure was not actively included in the updated “Europe’s Beating Cancer Plan” of the European Commission released in 2023 [5,6]. Nevertheless, countries should act and immediately adopt and realise strategies on managing HCV infection to prevent further development of HCC cases.

Since 2016, a strong, global public health response to HCV infection has been established by improving testing and access to DAAs, and by optimising the continuum of HCV care. Despite this enthusiasm, the continuum actually represents a downward cascade in the various steps of HCV care starting with low screening rates, followed by marked drops in confirmatory testing rates, and a steep decline in the rates of linkage to care and DAA treatment. In 2022, of the estimated 50 million people living with HCV infection globally, only 36% had been diagnosed and one-fifth had received curative treatment over the preceding 7 years [1]. So, while we have the tools to significantly decrease the burden of HCV infection, why do we not use them properly to scale-up the continuum of care so that the WHO targets are reached? Reaching the targets is not only crucial for the infected individuals, but for public health as well, since treatment also works as prevention of HCV transmission [7]. The message by WHO to its member states on how to achieve the elimination of HCV infection as a public health threat and prevent life-threatening conditions is clear and the recommendations are well established (Box).

Box **Steps recommended by the WHO to achieve the elimination of hepatitis C virus infections as a public health threat**

- estimate the burden of HCV infection,
- set the targets for HCV elimination,
- integrate viral hepatitis services into the existing healthcare system,
- increase access to HCV testing and treatment,
- promote equity,
- engage the community,
- improve surveillance and monitoring.

From [1].

Prevalence estimates of HCV infection are a critical metric to guide public health policies and monitor progress towards elimination goals in every country. Two studies by Hleyhel et al. in this issue of *Eurosurveillance* present recent prevalence estimates of HCV infection in the general adult population of two European countries: Estonia and Romania [8,9].

In Estonia, a high-income northern European country with 1.3 million population, the estimated 1.5–2% nationwide prevalence of chronic HCV infection, based on expert consensus in 2013, dropped to 0.8% in 2022. The latter result from a representative cross-sectional survey using prospectively collected samples, places Estonia among the countries with low HCV prevalence [8]. With the highest prevalence of chronic HCV RNA observed in men, in the age group 40–49 years, and in a certain geographic region, the data obtained likely reflect the historical trends in intravenous drug use (IDU) in Estonia, particularly since HCV predominance in males usually reflects HCV transmission by IDU. Since thus far, no national strategy on elimination of HCV infection has been endorsed in Estonia, the reduction in HCV prevalence can be partly explained by the death of infected people in older age groups, the introduction of DAAs, and a confirmed decrease in the size of the population of people who inject drugs (PWID) over the previous years.

On the other hand, in Romania, an eastern European country with a population of 19 million, which has only recently gained a high-income country status, the overall HCV seroprevalence decreased from 3.2% in 2006–2008 to 1.4% in 2020–2022. In the earlier nationwide study, estimates were significantly highest in females. The more recent estimates were obtained through an analysis of leftover samples with additional prospectively collected samples [9]. And with an estimated 0.9% prevalence of chronically infected individuals, Romania became a country of low HCV endemicity. This is most probably due to considerable efforts in scaling up testing for HCV, the availability of DAAs and improved access to treatment, all of which were endorsed in 2019 by the National Plan for Control of Viral Hepatitis [10].

The results from both studies are in line with the data from the European Union/European Economic Area countries obtained by the multiparameter evidence synthesis, showing that the estimated prevalence of chronic HCV infection in eastern European countries was 0.88%, compared with 0.27% in the western European countries [11]. Interestingly, in the Romanian study presented here, the HCV prevalence was highest in those aged 60 years and older, and slightly higher in females, with no predominance among PWID. This points out the high burden of past nosocomial mode of HCV transmission in contrast to other European countries, where PWID represent the population group driving the HCV infection epidemic with 72% of recently acquired infections being attributable to IDU [12].

Globally, one of the biggest challenges for achieving the WHO elimination goals for HCV infection as public health threat is inequity among the countries. The majority of high-income countries are committed to reaching the WHO targets by 2030 and several of them are on track, while the majority of middle- and low-income countries may never reach them [1]. In several of these countries, high costs of DAAs impose varying degrees of restrictions and limit the access to DAAs, leading to a situation of very low treatment coverage, particularly when combined with limited healthcare resources, and a lack of awareness [13]. However, a successful example of strong political awareness, will and commitment to HCV elimination at population level comes from Egypt, a country with financial constraints, yet with the highest HCV burden globally within a 60-million population in 2015 [14]. By setting up a massive population-level HCV screening to test nearly 50 million inhabitants in merely half a year, using generic DAAs and a test-and-treat strategy with a huge supporting infrastructure including 20,000 screening personnel, 7,000 screening sites, 1,000 mobile units for screening and treatment, and 165 treatment centres, in merely 5 years the prevalence of chronic HCV-infected individuals dropped from 10% to below 0.5% in 2020, making Egypt a low-endemic country on track to reach the HCV elimination.

Indeed, macro-elimination of HCV infection is difficult to achieve and for this reason many countries are implementing a micro-elimination strategy comprising systematic HCV screening and linkage to care within specific settings, regions or key populations presenting high HCV infection rates, such as people who use drugs (PWUD). Identifying the key populations for HCV infection is crucial to determine the need for specific interventions, while monitoring the trends in HCV prevalence may elucidate successful interventions or point to failures that need to be addressed to reduce the burden of HCV infection in certain high-risk groups.

The burden of HCV infection among PWUD is higher compared with the general population, even though the prevalence has decreased in recent years [15]. This is particularly relevant for PWID in whom HCV transmission is frequent because of ongoing risk behaviours [16,17]. A study by Ryan et al. in the previous issue of *Eurosurveillance* assessed the risk factors and temporal trends of active HCV infection in PWUD in a retrospective study conducted in a mobile screening unit in Madrid, Spain [18]. The data obtained confirmed that IDU is the most prominent risk factor for active HCV infection. The overall rate of active HCV infection among PWUD visiting the mobile unit notably decreased from 23.4% in 2017 to 6.0% in 2023, confirming the effectiveness of the applied prevention and treatment strategies. However, HCV infection remained high among those who injected drugs, highlighting the need for continuous interventions such as harm-reduction strategies, including needle and syringe programmes and opioid agonist treatment, as well as HCV treatment to reduce the burden of HCV infection in this high-risk group. Continuous monitoring of HCV prevalence trends among high-risk groups is crucial, and the use of simple point-of-care HCV testing and DAA therapy at the community level is of particular importance, especially when it is introduced by civil society organisations that can fill the gap in areas with marginalised and vulnerable groups that may be difficult to reach for traditional healthcare services [19,20]. Together with harm reduction programmes, the introduction of such strategies will allow a better control of HCV infection in this key population.

In addition to PWUD, monitoring and linkage to care remains an issue in other populations as shown to be relevant from the experience in Australia, a country strongly committed to eliminating hepatitis C as a public health threat. Prior to the study by Abbott et al. presented as well in the previous *Eurosurveillance* issue, there has been no active follow-up of notifications of individuals diagnosed with chronic hepatitis C by the Victorian Department of Health (DH) [21]. The pilot study aimed to evaluate the feasibility of follow-up by DH with diagnosing clinicians to assess and support linkage to care. More than 85% of diagnosing clinicians had provided appropriate follow-up care for their patients and identified the main barriers for patients to access treatment, whereas for the remaining diagnosing clinicians, missing contact information for doctors was a barrier to implementation, particularly in hospital settings. The study revealed the importance of piloting systems of referral and follow-up particularly for those diagnoses made at hospital level, and provided evidence for the feasibility and effectiveness of the use of surveillance systems to enhance access to care and treatment for hepatitis C. High proportions of lost to follow-up (LTFU) individuals with chronic HCV infection can endanger achieving the WHO goals to eliminate HCV infection as a public health threat. Thus, follow-up of notified cases could be a key tool in pursuing hepatitis C elimination. Such a micro-elimination strategy focused on LTFU cases showed promise in some European countries since re-engagement of LTFU individuals proved to be feasible and may lead to improving HCV confirmatory testing uptake and treatment outcome [22].

Taken together, much has been done on the way to eliminate hepatitis C as a public health threat, yet the speeds and the needs vary immensely among countries globally and within Europe. One size does not fit all. But regardless of the countries' income status or geographic location, it is of utmost importance to accelerate evidence-based activities, such as notifications promoting linkage to care, and putting HCV testing and treatment at the community level by rising awareness and using a simple, decentralised, and non-stigmatising approach, particularly for vulnerable populations. However, with no available HCV vaccine, setting up and empowering harm-reduction facilities and using treatment as prevention are currently the most effective prevention strategies on the way to HCV infection elimination. By eliminating HCV infections, we will also have gained the power to decrease the incidence of HCC, a severe disease with high fatality.

## Note

Mojca Matičič is Professor of Infectious Diseases and Epidemiology and Head of the Viral Hepatitis Unit and the STI Outpatient Service at the University Medical Centre in Ljubljana, Slovenia. She is a member and/or scientific advisor of international working groups on HCC and viral hepatitis at WHO, ECDC and ESCMID Global.

Maria Buti is Professor of Medicine and senior consultant at the Vall d’Hebron Hospital Universitari, Barcelona, Spain. She is a former President of the Spanish Association for the Study of Liver Diseases and former Chair of Public Health at the European Association for the Study of the Liver.

## References

[1] World Health Organization (WHO). Global health sector strategies on viral hepatitis, 2016-2021. Towards ending viral hepatitis. Geneva: WHO; 2016. Available from: https://www.who.int/publications/i/item/WHO-HIV-2016.06

[2] Popping S Bade D Boucher C van der Valk M El-Sayed M Sigurour O The global campaign to eliminate HBV and HCV infection: International Viral Hepatitis Elimination Meeting and core indicators for development towards the 2030 elimination goals. J Virus Erad. 2019;5(1):60-6. 10.1016/S2055-6640(20)30281-8 30800429 PMC6362901

[3] World Health Organization (WHO). Global hepatitis report 2024: action for access in low- and middle-income countries. Geneva: WHO; 2024. Available from: https://www.who.int/publications/i/item/9789240091672

[4] Dash S Aydin Y Widmer KE Nayak L . Hepatocellular carcinoma mechanisms associated with chronic HCV infection and the impact of direct-acting antiviral treatment. J Hepatocell Carcinoma. 2020;7:45-76. 10.2147/JHC.S221187 32346535 PMC7167284

[5] Vargas-Accarino E Higuera M Buti M Mínguez B . Hepatitis-C-related hepatocellular carcinoma, still a relevant etiology beyond a hepatitis C infection cure. Cancers (Basel). 2024;16(8):1521. 10.3390/cancers16081521 38672603 PMC11048451

[6] European Union (EU) Commission. Europe's beating cancer plan. Brussels: EU Commission; 2023. Available from:https://ec.europa.eu/commission/presscorner/detail/en/ip_23_421

[7] Olafsson S Tyrfingsson T Runarsdottir V Bergmann OM Hansdottir I Björnsson ES Treatment as Prevention for Hepatitis C (TraP Hep C) - a nationwide elimination programme in Iceland using direct-acting antiviral agents. J Intern Med. 2018;283(5):500-7. 10.1111/joim.12740 29512219

[8] HleyhelM GellerJ SadouA NaaberP KuznetsovaT VorobjovS Prevalence of chronic hepatitis C infection in the general population in Estonia: results from a national survey, July to December 2022. Euro Surveill. 2024;29(30):2300651.10.2807/1560-7917.ES.2024.29.30.230065139056201

[9] HleyhelM PopoviciO LeușteanM ReedS SadouA FuregatoM Prevalence of chronic hepatitis C infection in the general population of Romania: results from a national survey. Euro Surveill. 2024;29(30):2300663.10.2807/1560-7917.ES.2024.29.30.230066339056200

[10] Coalition for Global Hepatitis Elimination. The National Framework Plan for the viral hepatitis control in Romania, 2019-2030, coordinated by the Ministry of Health Romania (National Program). [Accessed: 28 Aug 2023]. Available from: https://www.globalhep.org/about/partner-programs/national-framework-plan-viral-hepatitis-control-romania-2019-2030

[11] Thomadakis C Gountas I Duffell E Gountas K Bluemel B Seyler T Prevalence of chronic HCV infection in EU/EEA countries in 2019 using multiparameter evidence synthesis. Lancet Reg Health Eur. 2023;36:100792. 10.1016/j.lanepe.2023.100792 38188273 PMC10769889

[12] European Centre for Disease Prevention and Control (ECDC). Monitoring of the responses to the hepatitis B and C epidemics in EU/EEA countries, 2023. Stockholm: ECDC; 2024. Available from: https://www.ecdc.europa.eu/en/publications-data/monitoring-responses-hepatitis-b-and-c-epidemics-eueea-countries-2023

[13] Marshall AD Willing AR Kairouz A Cunningham EB Wheeler A O’Brien N Global HCV and HIV Treatment Restrictions Group . Direct-acting antiviral therapies for hepatitis C infection: global registration, reimbursement, and restrictions. Lancet Gastroenterol Hepatol. 2024;9(4):366-82. 10.1016/S2468-1253(23)00335-7 38367631

[14] Hassanin A Kamel S Waked I Fort M . Egypt’s ambitious strategy to eliminate hepatitis C virus: A case study. Glob Health Sci Pract. 2021;9(1):187-200. 10.9745/GHSP-D-20-00234 33795369 PMC8087425

[15] Surey J Menezes D Francis M Gibbons J Sultan B Miah A From peer-based to peer-led: redefining the role of peers across the hepatitis C care pathway: HepCare Europe. J Antimicrob Chemother. 2019;74(Suppl 5):v17-23. 10.1093/jac/dkz452 31782500 PMC6883389

[16] Zibbell JE Asher AK Patel RC Kupronis B Iqbal K Ward JW Increases in acute hepatitis C virus infection related to a growing opioid epidemic and associated injection drug use, United States, 2004 to 2014. Am J Public Health. 2018;108(2):175-81. 10.2105/AJPH.2017.304132 29267061 PMC5846578

[17] Palmateer NE McAuley A Dillon JF McDonald S Yeung A Smith S Reduction in the population prevalence of hepatitis C virus viraemia among people who inject drugs associated with scale-up of direct-acting anti-viral therapy in community drug services: real-world data. Addiction. 2021;116(10):2893-907. 10.1111/add.15459 33651446

[18] Ryan P Valencia J Cuevas G Amigot-Sanchez R Martínez I Lazarus JV Decrease in active hepatitis C infection among people who use drugs in Madrid, Spain, 2017 to 2023: a retrospective study. Euro Surveill. 2024;29(29):2300712. 10.2807/1560-7917.ES.2024.29.29.2300712 39027941 PMC11258947

[19] Sheehan Y Cunningham EB Cochrane A Byrne M Brown T McGrath C A ‘one-stop-shop’ point-of-care hepatitis C RNA testing intervention to enhance treatment uptake in a reception prison: The PIVOT study. J Hepatol. 2023;79(3):635-44. 10.1016/j.jhep.2023.04.019 37116714

[20] Maticic M Pirnat Z Leicht A Zimmermann R Windelinck T Jauffret-Roustide M The civil society monitoring of hepatitis C response related to the WHO 2030 elimination goals in 35 European countries. Harm Reduct J. 2020;17(1):89. 10.1186/s12954-020-00439-3 33213481 PMC7678126

[21] Abbott M MacLachlan JH Romero N Matthews N Higgins N Lee A A pilot project harnessing surveillance systems to support clinicians providing clinical care for people diagnosed with hepatitis C in Victoria, Australia, September 2021 to 31 March 2022. Euro Surveill. 2024;29(29):2400028. 10.2807/1560-7917.ES.2024.29.29.2400028 39027939 PMC11258944

[22] Isfordink CJ van Dijk M Brakenhoff SM Kracht PAM Arends JE de Knegt RJ Hepatitis C Elimination in the Netherlands (CELINE): How nationwide retrieval of lost to follow-up hepatitis C patients contributes to micro-elimination. Eur J Intern Med. 2022;101:93-7. 10.1016/j.ejim.2022.04.024 35527178

